# Developing a Bayesian adaptive design for a phase I clinical trial: a case study for a novel HIV treatment

**DOI:** 10.1002/sim.7169

**Published:** 2016-11-27

**Authors:** Alexina J. Mason, Juan Gonzalez‐Maffe, Killian Quinn, Nicki Doyle, Ken Legg, Peter Norsworthy, Roy Trevelion, Alan Winston, Deborah Ashby

**Affiliations:** ^1^ Department of Health Services Research and PolicyLondon School of Hygiene and Tropical Medicine15‐17 Tavistock PlaceLondonWC1H 9SHU.K.; ^2^ Imperial Clinical Trials UnitImperial College London68 Wood LaneLondon W12 7RHU.K.; ^3^ Section of Infectious Diseases, Department of MedicineImperial College LondonLondonW2 1PGU.K.; ^4^ HIV i‐Base

**Keywords:** Bayesian adaptive designs, dose‐finding studies, continual reassessment method, fusion inhibitor, HIV clinical trials, phase I

## Abstract

The design of phase I studies is often challenging, because of limited evidence to inform study protocols. Adaptive designs are now well established in cancer but much less so in other clinical areas. A phase I study to assess the safety, pharmacokinetic profile and antiretroviral efficacy of C34‐PEG_4_‐Chol, a novel peptide fusion inhibitor for the treatment of HIV infection, has been set up with Medical Research Council funding. During the study workup, Bayesian adaptive designs based on the continual reassessment method were compared with a more standard rule‐based design, with the aim of choosing a design that would maximise the scientific information gained from the study. The process of specifying and evaluating the design options was time consuming and required the active involvement of all members of the trial's protocol development team. However, the effort was worthwhile as the originally proposed rule‐based design has been replaced by a more efficient Bayesian adaptive design. While the outcome to be modelled, design details and evaluation criteria are trial specific, the principles behind their selection are general. This case study illustrates the steps required to establish a design in a novel context. © 2016 The Authors. *Statistics in Medicine* Published by John Wiley & Sons Ltd

## 1 Introduction

Phase I clinical trials are typically set up to establish the safety of a proposed drug, study the pharmacokinetics (PK) and pharmacodynamics of this drug and to identify a dose which is suitable for taking forward to a further trial. These are first in human studies, usually involving small numbers of participants, but they are crucial in the development of new treatments. A poor trial design will increase the chance of discontinuing research into a promising drug prematurely or unnecessarily investing further resource in a drug. Additionally, a well‐chosen dose to take forward is essential for maximising the probability of making the correct decision about the drug at the end of any follow‐up study. Given the stakes, the choice of design is an extremely important part of setting up a phase I study.

The traditional approach for phase I trials is rule‐based, typically the ‘3+3’ design or variations 1, 2. These types of designs use prespecified rules to assign patients to dose levels, choose the recommended dose for the next study and do not make any assumptions regarding a dose‐response curve. For example, in a cancer setting where toxicity is the response, groups of three patients are treated starting at the lowest dose level. Escalation occurs if none of the patients experience a toxicity, and if more than one toxicity is observed, the trial stops. If one toxicity is observed, a further three patients are treated at the same level, and escalation then takes place if no further patients experience a toxicity, otherwise the trial stops. Dose escalation continues in this way until at least two patients among a cohort of three to six experience dose‐limiting toxicities. The recommended dose for the phase II trial is defined as the highest dose with less than two out of six toxicities.

However, while easy to implement, the operating characteristics of rule‐based designs tend to be unattractive 3. Not only can such designs lead to poor decision making regarding the future investigation of the drug but they may also expose unnecessary numbers of participants to inappropriate doses. The alternative to rule‐based designs is model‐based designs, which seek to model a dose‐response curve using all available information. Model‐based designs can be conveniently carried out within a Bayesian framework, and the first of these, the continual reassessment method (CRM), was introduced by O'Quigley *et al.* 4.

Continual reassessment method designs were mainly developed in the field of oncology, where the aim is to find a dose that will benefit the patient without causing an unacceptable amount of toxicity, known as the maximum tolerated dose (MTD). This requires defining an acceptable level of toxicity, the target toxicity level (TTL) and a dose‐toxicity curve. These methods assume that there is a monotonic dose‐response relationship between the dose and the probability of dose‐limiting toxicity (DLT) for patients treated at that dose. Since its inception, various modifications to the CRM have been proposed to address safety concerns, for example starting at a more conservative dose level, treating more than one patient at the same level and not skipping doses 5, 6, and Babb *et al.* 7 introduced an adaption of the CRM to reduce the risk of overdose, called escalation with overdose control.

While the CRM has primarily been used in cancer trials, its use has also been demonstrated in early phase stroke trials 8, and it has potential use in any area where finding the MTD or similar quantity is required 9. Further, Bayesian model‐based designs have been developed for phase I trials with dual endpoints, efficacy and toxicity, 10 and to allow for non‐monotonicity in the dose‐response curve 11. O'Quigley *et al.* 12 discuss adaptive designs for early dose‐finding studies in HIV‐disease, incorporating information on efficacy as well as toxicity. Their work was motivated by clinical trials designed to evaluate new antiretroviral treatments for children infected with HIV in general, rather than a specific trial. We are unaware of any published examples of the use of the CRM for phase I trials of the treatment of HIV infection that are to be implemented.

C34‐PEG_4_‐Chol is a novel peptide fusion inhibitor for the treatment of HIV infection. The funding application to the British Medical Research Council (MRC) for a phase I study to assess C34 was based on a simple dose‐ascending, rule‐based design, but incorporated a request for resources to investigate alternative designs. In this paper, we describe our experience of developing an adaptive design using the CRM in this novel context.

By documenting the process of the design development, we hope that others can learn from our experiences and be encouraged to explore the use of adaptive designs for their own studies. The main elements are described in the narrative of Sections 2–7. In Section 2, we introduce the C34 study, describe the initial and adaptive designs in Sections 3 and 4, respectively and detail the test scenarios used for evaluation in Section 5. The implementation, assessment of the relative merits of the different designs and results of the simulations are the subjects of Section 6. In Section 7, we describe the basis for choosing the final design, before concluding with a discussion.

## 2 Case study

Approximately 35 million people are infected with HIV worldwide 13. Left untreated, progressive immunosuppression ensues rendering HIV‐positive individuals vulnerable to opportunistic infections and malignancies and ultimately proving fatal within 10 years in a majority of cases 14. Improvements in combination antiretroviral therapy have resulted in the ability to achieve durable viral suppression, halt progressive damage to the immune system and lead to immune recovery. As a consequence, large cohort studies have shown that excess mortality rates in the HIV‐positive population have fallen from 40.8 per 1000 person‐years prior to 1996 to 6.1 per 1000 person‐years in 2004 to 2006 15. Similarly, dramatic increases in the life expectancy of those living with HIV have been observed over the same time frame, with one cohort study from the UK estimating life expectancy for individuals starting on antiretroviral therapy at 20 years had increased from 30.0 years if started between 1996 to 1999 to 45.8 years if started between 2006 and 2008 16.

There are now more older HIV‐positive individuals. The medical care of ageing HIV‐positive populations are complex for several reasons. Firstly, age‐related medical conditions appear to occur frequently and at a younger age in HIV‐positive subjects 17. Antiretroviral medication which can exacerbate such medical problems requires to be avoided. Secondly, the use of concomitant medication to manage these age‐related co‐morbidities is frequent and may interact with antiretroviral medication 18. Therefore, antiretroviral agents with few end‐organ toxicities and few drug–drug interactions are required to treat older HIV‐positive subjects.

There are six current classes of antiretroviral drugs, four of which target viral replication through inhibition of viral enzymes (reverse transcriptase, protease and integrase) and two of which block viral cell entry. Current standard of care comprises a combination of three agents, coming from at least two different classes. Many agents are associated with long‐term toxicities including metabolic, renal and bone toxicities and as such, may be challenging to use in older subjects with age‐related medical problems.

C34 is a molecule belonging to the fusion inhibitor agents, a class of antiretroviral used exceptionally rarely because of the requirement for twice daily subcutaneous injections and frequent injection site reactions but which remain highly attractive to further exploitation in view of their very low systemic toxicity profile and limited propensity for drug–drug interactions 19. By modification of the molecule to attach a cholesterol moiety and enhance solubility through pegylation, a derivative, C34‐PEG_4_‐Chol, has been developed which has been shown to be highly potent *in vitro* and is thought to possess potential as a once or twice weekly injectable agent 20. Definitive PK and efficacy profiling within a first in man study is a priority before phase II and III studies are undertaken.

Given the advantages of C34 outlined previously, we plan to run a dose and dose‐schedule finding phase I, first in man study to assess the safety, PK and pharmacodynamics of C34 in people with HIV infection. This will be a two‐stage, double‐blind, randomised, placebo‐controlled study. In stage 1, a single dose of C34 will be administered, while stage 2 will assess multiple‐dosing of C34. The population considered for this trial will be HIV‐1 infected male adults, in reasonably good health and not imminently requiring antiretroviral therapy. This places practical constraints on finding participants, as there is a small window for their recruitment, and with changing clinical practice, this window is becoming narrower. Further, there is a strong ethical argument for minimising recruitment, because participants receiving active treatment may develop resistance to fusion inhibitors, impacting the future use of this class of drugs for their treatment. Taking these considerations into account, along with the costs of the drug and running this type of study, a maximum of 42 participants will be enrolled across the two‐study stages. This number is considered sufficient to detect any common adverse events and to provide a useful amount of information on the PK profile.

In the first stage, the safety, tolerability and the PK profile of C34 will be assessed following administration of a single dose of C34. Additionally, the lowest dose which achieves concentrations 10 times above the IC_50_(IC_50_ = 0.0025*μ*g/mL) at the furthest time point after administration will be determined. We will refer to this dose as the ‘target dose’, and this will be the dose used in stage 2. A maximum of 28 participants will be enrolled in stage 1, with up to 20 participants receiving active drug and 8 receiving placebo. The use of placebo was to tease out drug‐related side effects, and does not impact on the statistical design, as the data for those patients is not pertinent, so we do not consider it further within this paper.

The aim of stage 2 is to assess the safety, tolerability and the PK profile of C34 following administration of multiple doses of C34. The dosing interval and dose to be administered will be determined upon review of safety and PK parameters from stage 1. A total of 14 participants will be enrolled, 11 receiving the active drug and 3 receiving placebo. Stage 2 will not be considered further in this paper, as the details of its design are dependent upon the outcome of stage 1.

## 3 Initial design

In the original funding application to the MRC, a dose‐ascending, rule‐based design was proposed for stage 1, consisting of four dose groups of seven participants, five receiving active drug and two receiving placebo according to random allocation, with each dose escalation subject to a satisfactory safety review of the results from the preceding dose group. These doses were chosen to include a lowest dose thought to be sub‐therapeutic, two middle doses thought to be around the range where efficacy may be seen and lastly the maximum dose that can be practically administered.

However, the application also stated that ‘prior to protocol submission to Ethics Committees further work will be performed … to simulate alternative approaches which may lead to more insightful understanding of the final data. For example, methods to characterise the dose‐response relationship will be assessed’. The intention was to use an adaptive design instead, provided this was likely to make more efficient use of the 20 subjects given active drug. Crucially, a request for statistical support to carry out simulation work to finalise the study design was included in the application.

The objective of the further design work was to explore whether the relevant parts of the dose‐response relationship could be more accurately characterised using an alternative design. The four dose group design was included in the simulations for benchmarking, with the doses specified as 10, 20, 40 and 80 mg. The maximum single dose we can administer in a reasonable volume is 80 mg. We will refer to this design as the ‘5+5+5+5’ design. The sample size was assumed fixed at 20 participants on active treatment as specified in the MRC funding application, and the implications of changing this were not investigated.

### 3.1 Limitations of initial design

A drawback of this design is that the doses are fixed in advance, and so no use is made of the accruing information as the trial progresses. As this is a first in man study, there is high uncertainty about the true dose‐response curve, and hence with this initial design, we may end up gathering much information on part of the curve that is not of particular interest. The trial's protocol development team, which included clinicians, statisticians, project managers and patient representatives (referred to as the ‘trial team’ henceforth), decided to develop an adaptive design that would enable them to maximise the relevant information gathered from a limited number of participants.

## 4 Development of adaptive design

To utilise the best features of an adaptive design without compromising safety, the trial team agreed that their alternative design would comprise three study periods: (i) an initial dose escalation and safety phase; (ii) an adaptive dose phase; and (iii) an optional final cohort phase. Period 1 will be similar to the 5+5+5+5 design, using the same doses but randomising two rather than five participants to the active treatment. All the administration of placebo will occur in this first period, leaving a further 12 participants to be administered active drug. Subject to the dose escalation phase indicating that the proposed dose range is safe, in period 2, 10 of the remaining participants will be allocated one of the doses under consideration, taking account of results from all previous cohorts. In period 3, optionally the remaining two participants will be given the dose selected at the end of period 2 for the multiple‐dosing stage, to allow improved estimation of the PK profile. Whether period 3 takes place will be determined by the Trial Steering Committee, with advice from the Independent Data Monitoring Committee (IDMC).

The benefits of the multiple periods are various. Not only does the first period enable the safety of the proposed drug range to be checked, it also mitigates the detrimental effects of an early unexpected outcome on the performance of the subsequent adaptive period^21^. The optional period 3 provides valuable flexibility. Further comment on safety issues is deferred to Section 6.3.

**Figure 1:**
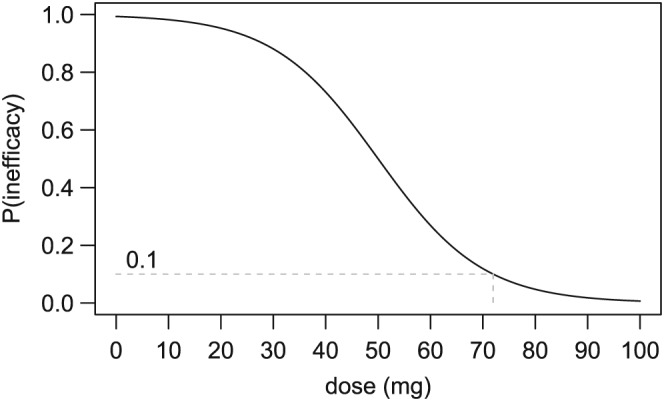
Dose‐inefficacy curve Illustration of a dose‐inefficacy curve for a model‐based trial design. If the target inefficacy level is 0.1, then the 70 mg dose will be chosen for the next cohort as it comes closest to yielding the desired probability of inefficacy. However, 80 mg will be the dose chosen to take forward as it is the lowest dose with probability of inefficacy below the target inefficacy level.

The design of period 1 is rule‐based and well defined, so the focus of debate for the trial team centred on the specification of the adaptive part, period 2. As discussed in the introduction, adaptive designs for phase I clinical trials typically use some variant of the CRM, which models a dose‐response curve assumed to be monotonic. Whereas in cancer trials, the relationship between dose and toxicity is modelled; for the C34 trial, we are interested in the probability of inefficacy given dose. Hence, a CRM will be used to model a dose‐inefficacy curve rather than a dose‐toxicity curve (Figure 1), and the response will be whether or not the participant experiences inefficacy rather than whether they experience a DLT. We assume that the probability of inefficacy decreases monotonically as the dose increases, in contrast to the assumption of a monotonically increasing dose‐toxicity curve for cancer studies.

**Figure 2:**
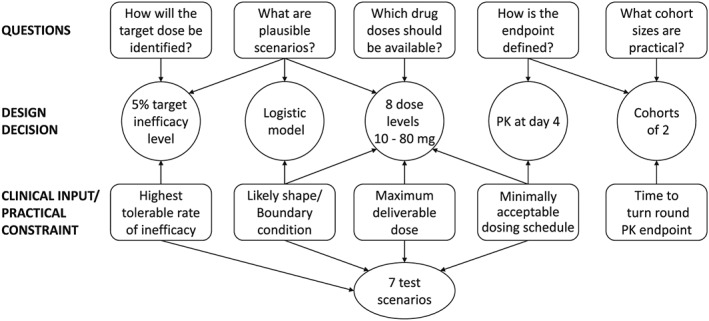
Interaction between key questions, features of the adaptive design and important clinical input and practical constraints.

While this superficially seemed like a straightforward application of the CRM, the trial team were required to determine the implementation details and the key questions needed to be addressed. Figure 2 shows five key questions, associated clinical input and practical constraints, and how they link with important design decisions. Although the questions are interlinked, we now consider each and their implications separately, starting with the most important.

### 4.1 How is the endpoint defined?

Inefficacy was defined as failure to achieve drug concentrations 10 times above the IC_50_ at the furthest time point after administration. Knowing that ideally the clinicians wanted a dose that achieved efficacy for all patients led to the realisation that the trial should be set up to explore the righthand tail of the dose‐response curve.

The ‘furthest time point after administration’ was also defined. Twice weekly dosing is considered the minimally acceptable dosing schedule, suggesting that our inefficacy endpoint should be calculated at day 4. However, weekly dosing is preferable, so it was agreed to build in some flexibility. At the end of period 1, a full PK analysis will be carried out using the first eight participants to determine whether there is evidence that the lower doses are effective up to day 4 and the higher doses are effective to day 7. With this information, a decision will be taken as to which endpoint is used in the adaptive period: we will initially use 7 days but would move to 4 days if the initial PK data suggest 7 days is not feasible. Consequently, the target dose is expected to be one of the higher doses, which while not impacting the simulation set‐up did influence the choice of test scenarios, so most have a target dose between 40 and 80 mg.

During the adaptive dose phase of the trial, the full PK analysis for previous cohorts of participants will not be available at the point that the decision on the dose for each cohort is made. Hence, the CRM is driven by a short‐term measure, PK for a single day.

### 4.2 How will the target dose be identified?

In cancer trials, a TTL is defined, but we define a target probability of inefficacy, which we analogously call the target inefficacy level (TIL). The TTL is typically set at levels in the range 20–30% for cancer trials. The choice of TIL is key in determining the dose selected for each cohort during the adaptive phase. One of the motivations for using an adaptive rather than standard design was to dose more participants at or close to the dose that will be taken forward to stage 2 of the trial. Hence, the requirement was to define a TIL such that the right tail of the dose‐inefficacy curve is adequately explored. It was decided to assess three options through simulation: TIL = 5%, 10% and 20% (5% was deemed the lowest level worth considering, and it seemed unlikely that anything higher than 20% would home in on the part of the curve of interest).

### 4.3 What are plausible scenarios?

This question feeds directly into the selection of the test scenarios, which are required for assessing the performance of the proposed designs (Section 5). However, it is also key to specifying the functional form of the CRM design. In particular, the chosen functional form needs to reflect plausible scenarios in terms of likely shape and specifically what happens at the extremes, for example when no drug is given (these are termed boundary conditions).

The CRM models a binary response, which we denote by *y*. For participant *i*, *y*_*i*_ is set to 1 if they experience inefficacy and 0 otherwise. This response is modelled using the Bernoulli distribution, *y*_*i*_ ∼ *B* *e* *r* *n* *o* *u* *l* *l* *i*(*p*_*i*_), where *p*_*i*_ is the probability that participant *i* experiences inefficacy. This probability is dependent upon dose, so to complete the model specification, we need to choose a suitable family of dose‐response curves.

In theory, the choice of the functional form of the dose‐response curve is quite wide, but in practice, only a limited number of one‐parameter and two‐parameter models have been used: the hyperbolic tangent model, the power model, the one‐parameter logistic model and the two‐parameter logistic model. One‐parameter models require less information to estimate, but may not accurately depict the entire dose‐response curve. They are, however, adequate for estimating the part of the curve around the target dose 22, which is our main interest. Given the size of the trial, after some early experiments with two‐parameter models, the choice was restricted to one‐parameter models.

Graphs showing families of curves were prepared and shown to the trial team. These included three variants of the one‐parameter logistic model (fixed parameter, *α*, set to 1, 3 or 5) and a set of power curves. The shape of the logistic models better reflected the clinicians expectations than the power curves. The chosen value of *α* in the logistic model determines the position of the intercept (probability of inefficacy when no drug is given). If no drug is given, then the patient must experience inefficacy. To ensure the probability of inefficacy at dose 0 mg is close to 1, the one‐parameter logistic model with *α*=5 was chosen, and *p*_*i*_ is modelled using *l* *o* *g* *i* *t*(*p*_*i*_) = 5 − *β* *d*_*i*_, where *d*_*i*_ is the dose given to participant *i*.

### 4.4 Which drug doses should be available?

With the PK modelling from animal studies, it is envisaged that below 10 mg in humans would be sub‐therapeutic if delivered twice a week, and 40 mg is expected to be the minimum therapeutic dose. Given this product is administered by subcutaneous injection, any dose can be administered up to the maximum reasonably deliverable dose. However, based on 80 mg being the maximum deliverable dose, it was agreed that eight dose levels would be available during the trial, at 10 mg intervals from 10 to 80 mg.

The dose levels must be mapped to the horizontal axis of the dose‐response curve. Various approaches to this task are possible, one of which is ‘backward fitting’ 22. Using this method and assuming that *β* = 1, standardised doses can be calculated as 5 − *l* *o* *g* *i* *t*(*P*_*d*_(inefficacy)). This is an inexact science, but plugging the probabilities from Scenarios 1–3 into this formula suggests that dividing the actual doses by 10 will map them to a sensible scale, and this is the mapping that underpins the simulation work (Scenarios 1–3 are the ‘likely scenarios’, described in Section 5).

### 4.5 What cohort sizes are practical?

The PK endpoint will be required to update the model between cohorts (see discussion in Section 4.1). Although the PK will only be for one day, the turn‐round will still introduce delay. The original CRM used cohorts of one patient, but most CRM designs now use cohorts of two to four patients. Garrett‐Mayer 22 recommends groups of two or three as providing the best balance between accurate estimation and safety. Bearing this in mind, and for reasons of practicality and confirmed by some early simulations, a cohort size of two was agreed for this trial.

## 5 Test scenarios

A set of scenarios was developed iteratively over a series of trial team meetings by specifying the probability of inefficacy at the eight dose levels, to reflect clinical expectations about the ‘true’ dose‐inefficacy curve of C34. Graphical representations of the scenarios were produced, plotted against the family of logistic curves underpinning the CRM. This gave an indication of how challenging each of the test scenarios would be for the proposed adaptive design.

Seven test scenarios were eventually chosen to cover a range of possible dose‐inefficacy curves, including two extreme scenarios and two scenarios which are considered unlikely to occur. The trial team considered 5% to be a tolerable rate of inefficacy for the dose selected for the multiple‐dosing stage (see Section 8.1 for rationale), so for each scenario, the dose specified as having the highest probability of inefficacy ⩽5% was defined as the target dose. The main features of these scenarios are summarised in Table 1.

**Table 1:** Summary of test scenarios.

Scenario	Target dose (mg)	Target used in period 1	Target on boundary*	Logistic shape	Monotonic shape
1: Standard PK model, 40 mg effective dose	40	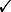	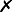	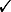	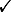
2: Standard PK model, 60 mg effective dose	60	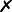	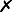	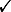	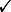
3: Standard PK model, 80 mg effective dose	80	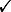	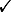	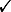	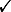
4: Standard PK model, no doses effective	none	n/a	n/a	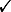	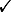
5: Standard PK model, all doses effective	10	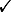	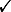	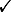	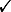
6: Standard PK model, partial efficacy with very low doses	60	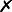	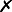	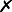	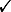
7: U‐shaped PK model	40	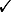	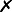	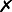	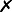

* The lower boundary is the lowest dose level (10 mg), and the upper boundary is the maximum dose level (80 mg).

Scenarios 1–3 have shapes which roughly follow a logistic curve, so the adaptive design is expected to perform well for these scenarios. Scenario 1 has a target dose in the middle of the available dose range, which has been selected as one of the four doses used in the safety phase (and by the ‘5+5+5+5’ design). Scenario 2 has a slightly higher target dose, which has not been selected for use in the safety phase. Scenario 3 tests the performance of the proposed model when the target dose is at the upper boundary of the available doses. Scenarios 4 and 5 are designed as ‘extreme’ scenarios, with Scenario 4 having no effective doses, and conversely for Scenario 5 all doses have inefficacy at 5% or below and so are considered effective.

**Figure 3:**
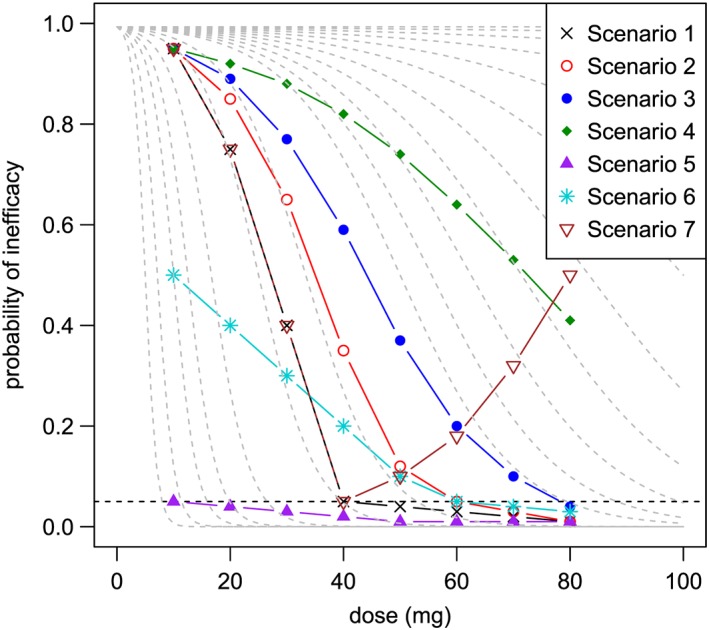
Test scenarios The dashed grey lines show members of the family of logistic curves specified for the proposed adaptive models. The horizontal dashed black line indicates the 5% level of inefficacy; target doses lie on or just below this line.

Scenarios 6 and 7 are the ‘unexpected’ scenarios, which are considered unlikely to occur. They are included to test the performance of the trial design if the shape of the dose‐inefficacy curve is unusual and does not approximate the shape of a logistic curve. Scenario 6 has a much shallower dose‐inefficacy curve than the other scenarios, with the same target dose as Scenario 2. Scenario 7 follows a reverse ‘J’ shape, mimicking Scenario 1 exactly for the first four doses before turning upwards. This type of shape could occur if the drug unexpectedly auto‐induces, giving rise to a situation where the probability of inefficacy initially decreases, reaching a tolerable rate before rising again. Scenario 7 is not thought at all likely to occur but is a good check of model robustness. Figure 3 provides a graphical representation of the specification of all these scenarios.

## 6 Simulation

Simulations were run and were gradually refined as decisions on various features of the design and test scenarios agreed. For example, before the cohort size decision was taken, some early simulations compared using cohort sizes of both one and two. Participants on placebo were ignored for the purposes of these simulations. In this section, we describe the final set of simulations. At this stage, the two main outstanding questions concerned the most appropriate value of the TIL and how to select the dose to take forward to stage 2.

### 6.1 Description of final set of simulations

The final set of simulations were run to investigate and compare the performance of the 5+5+5+5 design and three variants of the adaptive design with the TIL set to 5%, 10% and 20%, which we call *5% TIL*, *10% TIL* and *20% TIL*, respectively. For each scenario, 1000 realisations of the C34 trial were generated using each design. The 1000 realisations for one adaptive design for one scenario took approximately 10 h to complete on a laptop computer with an Intel(R) Core(TM) i7‐2620 CPU @ 2.70 GHz processor and 8 GB of RAM.

A rule‐based approach to selecting the dose to take forward to stage 2 was implemented for all four designs. This approach closely aligns with the original thinking of the clinicians but is inconsistent with the use of a model to select doses during the adaptive phase. Hence for the adaptive designs, a model‐based approach was implemented as an alternative. The two approaches are defined as follows:

**Rule‐based criteria:** select the lowest dose administered during periods 1 and 2, such that there are no failures at this dose or any higher doses. (The choice of dose is restricted to the 10, 20, 40 and 80 mg doses which are used in the dose escalation and safety phase, and any other doses used in the adaptive dose phase.)
**Model‐based criteria:** select the lowest dose with probability of inefficacy below the TIL. (This contrasts with the choice of dose for the next cohort during the adaptive phase, which is the dose with the probability of inefficacy closest to the TIL. It reflects the belief that selecting too low a dose is worse than selecting too high a dose.)

In reporting the simulation results, the words ‘rule’ and ‘model’ are used to indicate which criteria was used.

### 6.2 Implementation

The algorithm to run the simulations was implemented using the r software, version 3.0.1 23 with calls to WinBUGS 24, 25. An outline of the algorithm, including the code for the WinBUGS model is provided as an Appendix. The decision rule for selecting the dose for the next cohort is based on posterior probabilities as advocated by Ji *et al.* 26. The WinBUGS model was run with two chains, initialised using diffuse starting values, to obtain 10 000 samples for posterior inference, after discarding the first 1000 iterations from each chain as burn‐in.

**Figure 4:**
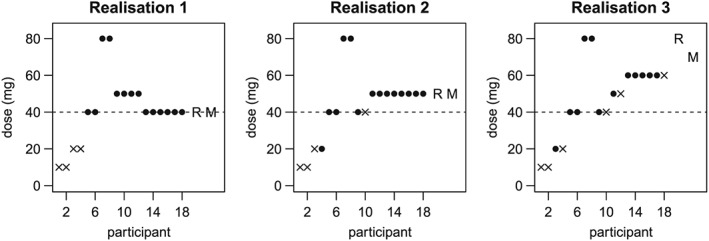
Example realisations of Scenario 1 simulation with the 5% TIL 5% TIL design Crosses and filled circles indicate ineffective and effective responses, respectively. The dashed line indicates the target dose, and the letters ‘R’ and ‘M’ indicate the dose chosen to take forward to Stage 2 using the rule‐based criteria and model‐based criteria, respectively.

To demonstrate how the adaptive designs work, the first three realisations of the Scenario 1 simulation using the *5% TIL* design are shown graphically in Figure 4. The dose allocated to each participant is plotted in the order they entered the trial. Crosses and filled circles indicate ineffective and effective responses, respectively. The dashed line indicates the level of the target dose, and the letters ‘R’ and ‘M’ indicate the dose recommended to take forward to the multiple‐dosing phase using the rule‐based criteria and model‐based criteria, respectively.

The positions of the first eight participants are always the same, as specified for period 1; however, the dose level of subsequent participants varies dependent upon the previous results. For the first realisation (left plot) where there were two ineffective responses at both 10 and 20 mg, but two effective responses at both 40 and 80 mg, participants 9 and 10 are given a 50 mg dose. However, for the second and third realisations (centre and right plots), which differ in having one effective response at 20 mg, these participants are allocated the 40 mg dose. The dose suggested for stage 2 is the same regardless of the basis of the decision for the first two realisations, but is different for the third realisation, reflecting the requirement of the rule‐based criteria to choose only doses which have been previously allocated.

**Figure 5:**
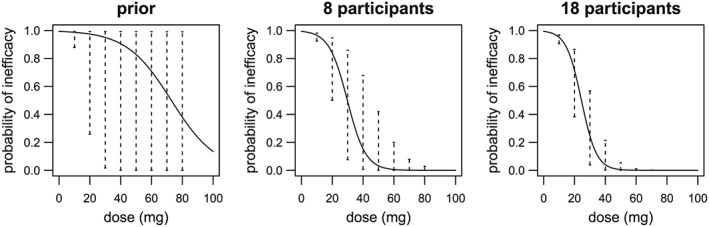
Change in estimated dose‐inefficacy curve as trial progresses (first realisation from 5% TIL 5% TIL simulations for Scenario 1) The dose‐inefficacy curve is calculated using the median of the posterior distribution of *β*. The dashed vertical lines indicate the 90% credible intervals for the probability of inefficacy at the possible doses.

The reduction in the uncertainty about the dose‐inefficacy curve as a trial progresses is demonstrated in Figure 5, using the first realisation from the *5% TIL* simulations for Scenario 1 as an example. The plots all show the dose‐inefficacy curve as a solid line, with dashed vertical lines indicating the 90% credible intervals for the probability of inefficacy at the possible doses. The dose‐inefficacy curve is calculated using the median of the posterior distribution of *β*. From the left plot, calculated before the trial begins, we see that the prior is vague as intended, with the 90% credible intervals for most doses covering the whole probability range from 0 to 1. As the trial proceeds, and data from the participants are combined with the prior, the position of the dose‐inefficacy curve changes, and the 90% credible intervals shrink (the positions after period 1 and at the end of period 2 are shown). From the posterior distribution of the probability of inefficacy of the chosen dose, based on the vague prior and data from 18 participants, there is a 90% chance that the probability of inefficacy for the 40 mg dose lies between 0% and 21% (90% credible interval), and its most likely probability of inefficacy is 4% (based on the median of the posterior distribution).

### 6.3 Measures of evaluation

The concerns of the HIV clinicians for this trial may differ from those often addressed in phase I studies in other disease areas. For instance, in cancer trials, not only is it important to correctly identify the MTD but also to minimise the number of subjects who are given a sub‐therapeutic drug dose or experience a DLT. In this trial, toxicity is not expected to be a major issue nor is under‐dosing a concern in this context. However, adverse events will be monitored and carefully reviewed by the IDMC (stopping criteria based on adverse events are detailed in the trial protocol). Indeed, any safety issues encountered are likely to be trial limiting and can override the trial design; while safety is a deal breaker here, the trial was planned on the working assumption that significant safety issues were unlikely to arise. For this reason, we formally concentrate on the PK and the priorities are the following:

- maximising the probability of selecting the target or higher dose to take forward;
- minimising the probability of wrongly rejecting all doses and not proceeding to stage 2;
- treating enough patients at the dose selected to take forward to allow an adequate PK analysis at this dose level.

As a result, the set of operating characteristics of interest is somewhat different to the cancer setting.

Using simulation, we assess the following:

1. The probability of selecting each dose (or no dose) for stage 2;
2. The number of subjects allocated each dose;
3. The number of observed ineffective events;
4. The number of subjects treated at doses below the target dose.

For each trial design, these measures are presented as means calculated over that design's set of realisations. We focus primarily on the first of these, also paying attention to the second. Operating characteristics 3 and 4 are of little interest in the context of the C34 trial but are included for completeness as they could be important for later phase HIV trials and in other therapeutic areas. A detailed table for each scenario, providing information about all four of these operating characteristics, was produced. Table 2 is for Scenario 1, and the tables for the other scenarios are provided as supplementary information.

**Table 2:** Operating characteristics under Scenario 1 (based on 1000 realisations).

	Dose									*N*	*N* below
	None	10 mg	20 mg	30 mg	40 mg	50 mg	60 mg	70 mg	80 mg	ineffective‡	target§
True inefficacy		0.95	0.75	0.40	0.05	0.04	0.03	0.02	0.01		
5+5+5+5											
*N* per dose†		5.00	5.00		5.00				5.00	8.82	10.00
*P* (selection)* – rule	0.05	0.00	0.00		0.73				0.22		
5% TIL											
*N* per dose†		2.00	2.01	0.72	7.10	2.76	0.82	0.02	2.56	4.20	4.74
*P* (selection)* – rule	0.02	0.00	0.00	0.07	0.48	0.20	0.12	0.01	0.09		
*P* (selection)* – model	0.01	0.00	0.00	0.00	0.52	0.28	0.15	0.02	0.02		
10% TIL											
*N* per dose†		2.00	2.03	1.58	8.59	1.26	0.33	0.05	2.16	4.55	5.61
*P* (selection)* – rule	0.02	0.00	0.00	0.01	0.56	0.18	0.03	0.01	0.20		
*P* (selection)* – model	0.00	0.00	0.00	0.01	0.62	0.30	0.04	0.02	0.00		
20% TIL											
*N* per dose†		2.00	2.12	4.48	6.94	0.38	0.05	0.00	2.03	5.73	8.60
*P*(selection)* – rule	0.01	0.00	0.00	0.01	0.69	0.06	0.00	0.00	0.22		
*P* (selection)* – model	0.00	0.00	0.00	0.10	0.84	0.06	0.01	0.00	0.00		

values for each trial design are means calculated over that design's set of realisations.

* the probability of selecting each dose (or no dose) to take forward to stage 2.

† the number of subjects allocated to each dose.

‡ the number of observed ineffective events.

§ the number of subjects treated at doses below the target dose.

However, the detailed information in these tables is difficult to absorb. This led to discussion within the trial team about the best way to summarise these operating characteristics using a clear pictorial representation. Focussing on the first operating characteristic, the clinical team were primarily interested in the probability of choosing a ‘good dose’ when there is a target dose or the probability of not selecting any dose when there is no target dose. Four summary measures were defined using different definitions of a ‘good dose’:

SM1: Probability of selecting the target dose or any higher dose;
SM2: Probability of selecting the target dose or a dose 10 mg higher;
SM3: Probability of selecting the target dose;
SM4: Probability of selecting the target dose or a dose 10 mg higher or lower.

**Figure 6:**
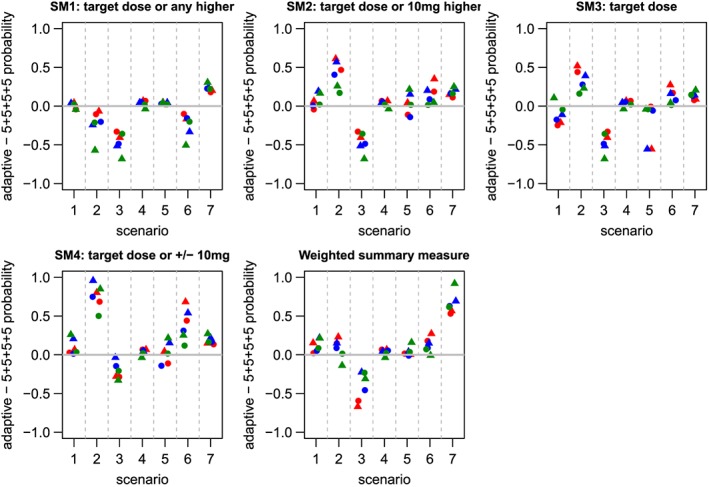
Summary measures of design performance: difference in probability of choosing a ‘good dose’ compared with the standard design A positive value indicates that the adaptive design performed better. For Scenario 4 which has no effective doses, the difference is in the probabilities of not selecting any dose. The *5% TIL*, *10% TIL* and *20% TIL* designs are represented by red, blue and green symbols, respectively. Circles indicate rule variants and triangles indicate model variants.

The trial team concluded that the most helpful presentation of these summary measures was a direct comparison of the adaptive designs with the 5+5+5+5 design (Figure 6). In this set of graphs, a positive value indicates that the adaptive design performed better than the 5+5+5+5.

In choosing a design for this study, the prime consideration is selecting a dose to take forward to the multiple‐dosing stage which maximises the chance of success. As such, choosing a dose that is lower than the target dose would not be acceptable to the clinicians, and hence the first summary measure is considered the most important.

Additionally, we calculated a weighted summary measure (WSM), based on SM1, which increasingly down‐weights the probability of choosing a dose as its distance from the target dose increases and penalises for failing to select any dose. Specifically, when there is a target dose, WSM is calculated as

$$p(d_{t})+\overset{max(8,t_{d}+4)}{\underset{i=t_{d}+1}{\sum }}p(d_{i})\times \left(1-\frac{d_{i}-d_{t}}{5}\right)-2p(\text{no dose}),$$

where *d*_*t*_ is the target dose divided by 10, and *p*(*d*_*t*_) is the probability of selecting the target dose. For example, if the target dose is 60 mg, then this measure will be calculated as

p(dose 6)+0.8p(dose 7)+0.6p(dose 8)−2p(no dose).

Alternatively, when there is no target dose, it is set to the probability of not selecting any dose as for the other summary measures. WSM is also shown in Figure 6.

**Figure 7:**
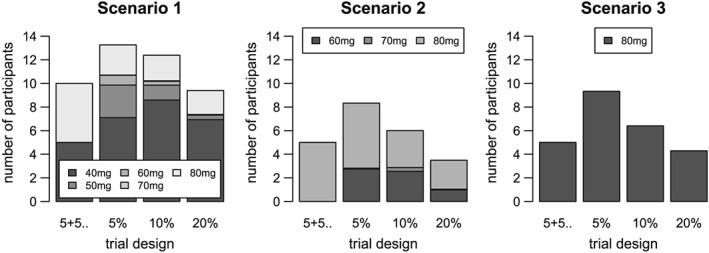
Number of participants given the target and higher doses: Scenarios 1–3 The bars indicate the number of participants given the target or higher doses. The target dose is shown at the bottom of the stack, and the dose furthest away at the top. The four trial designs shown are 5+5+..=5+5+5+5, 5%=5% TIL, 10%=10% TIL and 20%=20% TIL.

Figure 7 indicates the number of participants given the target or higher doses for Scenarios 1–3, with the target dose shown at the bottom of the stack. In this plot, the comparison is not precisely like with like, as the 5+5+5+5 has already dosed 20 participants while the adaptive designs still have the option of dosing two more participants. However, this provides evidence that the *5% TIL* and *10% TIL* adaptive designs allocate more participants to the doses of interest than the 5+5+5+5.

### 6.4 Results

Looking at the different summary measures across the seven scenarios, it is evident that no one design is the clear ‘winner’, rather the ‘best’ performing design varies according to the scenario and operating characteristics considered. However, a few overall findings emerge. Compared with the 5+5+5+5 design, the adaptive designs treat less participants at the lowest doses (10 and 20 mg) when the target dose is at a higher level. This is less ‘wasteful’ of participants because more PK information can be obtained in the part of the dose‐inefficacy curve of interest. In terms of selecting a ‘good’ dose, the 5+5+5+5 design does better than all the adaptive designs when the target dose is at the upper boundary, that is the maximum dose level, regardless of the definition of a ‘good’ dose (Scenario 3). However, when the target dose is not one of the doses administered in the 5+5+5+5 design, the adaptive designs do better unless equal credit is given to selecting the target dose and any higher dose (Scenario 2). For a non‐boundary target dose included in the 5+5+5+5 design, the differences between the two options are smaller, the better option being more sensitive to the chosen summary measure (Scenario 1).

Turning attention to the less likely scenarios, all the designs correctly selected no dose for Scenario 4 a very high proportion of the time. For Scenario 5, where the target dose is at the lower boundary, using the rule‐based criteria, the 5+5+5+5 and the adaptive designs select the target dose just over 50% of the time. The adaptive designs treat a lot of participants at 20 mg, and using the model‐based criteria select the 20 mg to take forward with high probability, particularly for *5% TIL* and *10% TIL*. In the more challenging Scenario 6, apart from for SM1, the adaptive designs outperform the 5+5+5+5 design. No design does well for Scenario 7, hardly surprising because it conflicts with the design assumptions.

Comparing the three adaptive designs for SM1 visually (Figure S1, supplementary material), the *5% TIL* design generally outperforms its equivalent *10% TIL* and *20% TIL* designs, and the *10% TIL* design usually does better than its equivalent *20% TIL* design. The situation is rather less clear‐cut and is scenario dependent for the other summary measures.

Again, no clear‐cut pattern emerges from a visual comparison of the rule‐based versus model‐based criteria (Figure S2, supplementary material): the better option depends on the scenario and summary measure considered as well as which TIL the design uses. In general, for the *5% TIL* and *10% TIL* designs, the model‐based criteria does at least almost as well as the rule‐based criteria. For the *20% TIL,* there are more instances of the rule‐based criteria doing best, particularly for Scenario 3. However, when the trial is run on real participants, both criteria can feed into the decision about the dose to be used in stage 2 of the study, as well as the detailed PK analysis.

Ideally, our analysis of each design would be based on more than 1000 realisations, but running time was a constraint. The uncertainty in the results was assessed by repeating the simulation for the *5% TIL* design for Scenario 1 four times. The range of the summary measures across the five runs was at most 5%, with a slightly larger range for the weighted summary measure which is on a different scale.

## 7 Choosing the final design

The results from Section 6.4 highlight that there is no optimal design and each of the summary measures reflects a different aspect, so we returned to the clinical imperatives. The clinicians in the trial team summarised the clinical advantages and disadvantages of an adaptive design as follows:

Advantage 1: More likely to select appropriate dose for schedules not covered by the 5+5+5+5 design (Scenario 2; *5% TIL* and *10% TIL* designs).
Advantage 2: More subjects administered optimal dose in settings where optimal dose is covered by the 5+5+5+5 design (Scenario 1; *5% TIL* and *10% TIL* designs).
Advantage 3: More subjects administered the optimal dose where the maximum dose is the optimal dose (Scenario 3; *5% TIL* design).
Advantage 4: Although less likely to select a dose where the maximum dose is the optimal dose (Scenario 3), in this situation, the study team are likely to override the adaptive design (i.e. if results look as if the maximum dose is the only option the Trial Steering Committee and IDMC would assess if further subjects should be recruited and administered this dose). Overriding study design would not occur with 5+5+5+5 design.
Neutral: Very little difference in dose selected between designs for scenarios where the optimal dose is covered by the 5+5+5+5 design (Scenario 1).
Disadvantage: Less likely to select a dose where the maximum dose is the optimal dose (Scenario 3). However, this disadvantage may be mitigated by advantages 3 and 4.

On the basis of the aforementioned clinical interpretations of the results presented in Section 6.4, the trial team concluded that overall, there appears to be clinical advantages in selecting the adaptive study design with 5% TIL.

## 8 Discussion

In this paper, we have outlined the steps needed to design an adaptive study in a novel context and applied them to design a study of a new antiretroviral compound. We paralleled a conventional CRM, developing analogous terminology to allow the parallels to be clearly seen. In this final section, we reflect on some of the important decisions required in this process, tease out some keys messages to different trial team members and comment on possible avenues of future research before concluding.

### 8.1 Reflections on design decisions

In Section 6, we described the final set of simulations which underpinned the choice of adaptive design. However, these simulations went through many iterations and are far removed from those initially run in just about every aspect. To get to this point was a slow evolution, as the trial team gradually homed in on the key areas and chipped away at the outstanding questions.

#### 8.1.1 Choice of endpoint

A clear understanding of the endpoint driving the study was crucial to making progress, as it impacted the answers to all the other questions. Although ‘inefficacy’ was the intended endpoint, its precise definition was not obvious, as discussed in Section 4.1. The driver of this study is to establish whether the drug can be used twice weekly or weekly, but the operational definition of that is critically dependent upon the data available. For example, if it had been logistically possible to have full PK data available in real time, then the endpoint would be defined differently.

#### 8.1.2 Target dose identification

Recall that we defined the target dose as the lowest dose which achieves concentrations 10 times above the IC_50_ at the furthest time point after administration. The clinical inclination was that this should be true for all patients, that is the probability of inefficacy should be 0%. However, working with 0% is not compatible with using a logistic model. The target dose is to be used for the follow‐up multiple‐dosing stage of this trial. For the purpose of setting up the test scenarios, we defined the target dose to be the lowest dose with probability ⩽5%. Given this is chosen from a discrete set of doses at 10 mg intervals, for the simulations, this will result in a target dose with a very low probability of inefficacy, which was considered reasonable for simulation purposes. There was discussion about how the target dose would be identified at the end of each realisation of the simulations, revolving around the pros and cons of using a rule‐based versus a model‐based criteria. Hence, the results of the simulations were reported using both criteria. Ultimately, we realised that this discussion was somewhat redundant, as both types of criteria could feed into the decision when the trial is run for real, along with more detailed information from a full PK analysis.

The choice of the TIL, which forms part of the specification of the CRM is a separate issue to the method of identification of the dose for the multi‐dosing stage, although it is clearly related. The key requirement in setting the TIL is to generate suitable information for making a decision about the dose for stage 2, that is to ensure that the right tail of the dose‐inefficacy curve is adequately explored. The choice impacts directly on the dose selected for the next cohort during stage 1, and because we wish to explore doses in the vicinity of the target dose, we select the dose with probability of inefficacy closest to, rather than below, the TIL. The final decision about the value of the TIL was based on simulation results which pointed to the 5% TIL, consistent with the 5% cut‐off used for selecting the target dose in the test scenarios. In fact, there was not very much difference between the 5% and 10% options, as the use of a discrete set of doses means that the chosen dose is often identical.

The simulation process brought home the interdependence of these two decisions. An interesting point emerged when considering Scenario 3, where the target dose was the maximum. Here, the 5% TIL resulted in more participants being administered the top dose than the other designs under consideration. While this provides more PK information for this dose, it also increases the probability of at least one participant experiencing an inefficacy and the target dose identification criteria suggesting there is no viable dose.

#### 8.1.3 Other decisions

Other features of the CRM design that needed to be chosen included the functional form, dose levels and cohort size. Besides these, there were a lot of decisions which were presented to the trial team but were beyond the scope of this paper. Model variants we decided not to pursue but were incorporated into interim sets of simulations, included using a two‐parameter logistic model as the functional form (added computational complexity, without altering the results substantially), different sets of dose levels (adjusted to comply with maximum reasonably deliverable dose) and a cohort size of one (less practical than a cohort size of two). The scenarios used to test the different CRM variants were also subject to continual revision, as they needed to take account of almost all the clinical inputs and practical constraints.

### 8.2 Messages

The development of the C34 trial design resulted from close collaboration between all the members of the trial team. This paper is largely written from the statisticians' perspective, however the statisticians' could not have developed the proposed design in isolation but were dependent on input from the other team members throughout the process.

#### 8.2.1 To statisticians

From the statisticians' viewpoint, the key was properly understanding the drivers of the study. Simulation skills were required, but the research could be carried out using standard tools available in free software. To maximise progress at the trial group meetings, great care was taken in the presentation of the latest simulations to the other group members. This worked best by synthesising the material onto a set of slides, starting with a recap of what had previously been agreed to make sure all team members were up to date. Results of the latest simulations were then shown, highlighting new elements. Graphical presentations were most easily understood and benefited greatly from feedback from the non‐statisticians in the team. The final part of the statisticians' presentation was a series of questions about outstanding issues. This led to discussion about the next steps required. We took the sample size as given, and graphs such as Figure 5 indicate that reasonably precise information is likely to emerge from this.

As pointed out by a reviewer, a CRM‐type design can focus participants on a single dose to the exclusion of generating safety and PK data across the range of doses under investigation. To mitigate this, we have chosen a ‘hybrid’ design, which incorporates a dose‐ascending phase to generate some data over the entire dose range followed by a CRM which will focus around the most promising dose. We believe this flexibility is key to enabling best use of the limited number of participants available for this trial.

#### 8.2.2 To non‐statisticians

An important message to clinicians is that they need to fully engage in the process of developing a trial design and cannot just delegate this to a statistician. The study design links to more elements of the trial protocol than just the sample size calculation. They need to be able to provide clarity on key issues such as endpoints and be prepared to challenge the statisticians. Initially, the non‐statisticians were keen to use hard rules to select the next drug dose but over time as successive sets of simulation results were explained, developed an understanding of the advantages of using probabilistic models to make the decision instead. Importantly, they accepted that the statistical models incorporated uncertainty and could not guarantee a correct answer. This led to a lot of thinking and discussion about what types of mistakes they could live with and what types they found totally intolerable. Ultimately, the fine‐tuning of the model was a result of a series of trade‐offs.

#### 8.2.3 To trial teams

The whole process of developing the adaptive design and agreeing its final detail was time consuming, taking over a year in elapsed time. It also required a considerable time commitment from the whole trial team in terms of attending regular meetings, which were held on an approximately monthly basis. In between these meetings, simulations were run and analysed by the team's statisticians. This level of resource allocation was made possible by the MRC, who allowed the cost of the workup of the design to be written into the grant. Allowing sufficient time and including adequate financial and personnel resources at the funding and planning stage were imperative for the design development. This aspect of trial workup was carried out in parallel with the development of other aspects of the protocol, design of the data collection system and associated animal toxicology studies. However, as expertise is built, this type of trial design could be worked up in a shorter time frame if necessary, but this would require careful planning.

### 8.3 Future research

Clinically, the next steps are implementing the identified adaptive design and running the trial, which will in turn inform the design of future trials. Statistically, a useful follow‐up would be to try these steps in designing future trials, to develop guidance such as checklists. Further aspects could be explored by additional simulation, for example the implication of different dose spacings, other dose level mappings and alternative priors. The implications of having fuller PK data available in real time and how this could be incorporated into the modelling, specifically through adapting the definition of the endpoint, could be assessed. We could consider refinement of the methods of presenting simulation results to clarify their implications. Further, where there is flexibility in the choice of the sample size, modifications could be made to the simulation process.

### 8.4 Conclusion

While much of the detail we have described is specific to this trial, many of the general principles followed are widely applicable. In going through this process ourselves, we have identified some steps which will be helpful to teams working up other early phase clinical trials. In particular, we stress the importance of good communication between all members of a trial team, and how developing the trial design is integral to the whole trial workup and should not be carried out in isolation.

The development of the chosen adaptive design involved much work and commitment, but we consider the effort justified. In this trial, an untried drug is to be given to individuals already diagnosed with a potentially life‐threatening disease, and we owe it to the participants to maximise the scientific information gained from the study. While we can rerun simulations many times if the design is not quite right, we do not want to be rerunning such a study on live patients.

## Supporting information

Supporting info item

## Appendix A Algorithm

### A.1

The algorithm used for each simulation is as follows:

Step 1: Assume a vague prior for *β*(exponential(1) distribution).
Step 2: Observe inefficacy outcomes for eight participants given active treatment in period 1.
Step 3: Update *β* by computing its posterior distribution, which is obtained by multiplying the prior and the likelihood. After *n* participants the likelihood is given by $L(\beta ;d,y)\propto \overset{n}{\underset{i=1}{\prod }}p{\left(d_{i}\right)}^{y_{i}}{\left[1-p(d_{i})\right]}^{1-y_{i}},$ where *d*_*i*_ and *y*_*i*_ are the dose level and inefficacy outcome for participant *i*(*y*_*i*_ is set to 1 for inefficacy and 0 otherwise). Compute also the posterior distributions of quantities required for selecting the dose for the next cohort (Step 4).
Step 4: Following Ji *et al.* 26, treat the next cohort of participants at dose *j* with the largest posterior probability $P[abs(p_{T}-p(d_{j}))=\underset{i}{\min}(abs(p_{T}-p(d_{i}))|data],$ where *p*_*T*_ is the target probability of inefficacy and *p*(*d*_*i*_) is the probability of inefficacy for dose *d*_*i*_, that is the dose with the highest probability of being closest to the target dose. (Here, *a* *b* *s*() represents the absolute value.) Observe the inefficacy outcomes.
Step 5: Update *β* and other quantities as described in Step 3.
Step 6: Repeat Steps 4–5 until 10 participants have been given drug in period 2.
Step 7: Select dose to be taken forward to Stage 2 in two different ways: Rule‐based decision: select the lowest dose administered during periods 1 and 2, such that there were no failures at this dose or any higher doses; Model‐based decision: select the lowest dose with probability of inefficacy below the target inefficacy level.

## Appendix B WinBUGS model

### B.1

The code for the WinBUGS model used in the simulations is shown here.

## Appendix C Contribution of authors

### C.1

Alexina J. Mason led the statistical aspects of the designs, building on the work of Juan Gonzalez‐Maffe, and wrote the initial draft of the paper, revising it in the light of comments from others. J. G. M. initiated the development of the statistical aspects of the designs. Killian Quinn, Nicki Doyle, Ken Legg, Peter Norsworthy, Roy Trevelion, Alan Winston and Deborah Ashby are on the Trial Management group and participated in a series of meetings to develop the designs, and the criteria for making the final choice. In addition, AW and DA developed the original study proposal and are grant‐holders of the MRC grant that funds it. They have overseen this part of the work. All authors have commented on drafts and approved the final version of the manuscript.
