# Supplementary material for: Developing a Bayesian adaptive design for a phase I clinical trial: a case study for a novel HIV treatment
Source: Stat Med. 2016 Nov 27;36(5):754–71. doi: 10.1002/sim.7169 (PMC5412923; doi:10.1002/sim.7169)
Supplement: Supplementary file 1 — Supporting info item [file SIM-36-754-s001.pdf]

Developing a Bayesian adaptive design for a Phase I clinical trial: a case study for a novel HIV treatment

Supplementary Information

Figure S1: Comparison of different target inefficacy levels

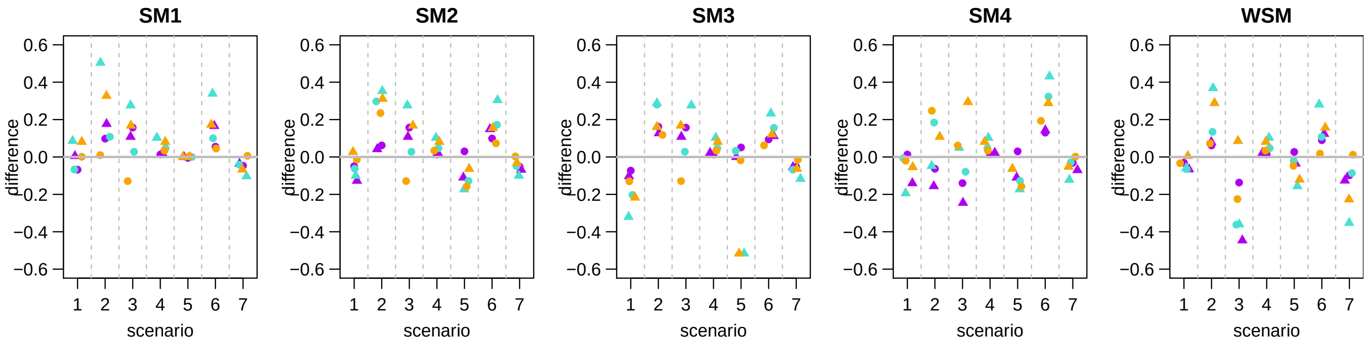

Comparisons between (i) 5pc designs and 10pc design (5pc-10pc), (ii) 5pc designs and 20pc designs (5pc-20pc), and (iii) 10pc designs and 20pc designs (10pc-20pc) are represented by purple, turquoise and orange symbols respectively. For comparison (i) positive values indicate that the 5pc design performed better than its 10pc counterpart; similarly for comparisons (ii) and (iii) positive values indicates the 5pc and 10pc designs respectively outperform the 20pc design. Circles indicate rule variants and triangles model variants.

Figure S2: Comparison of 'rule' and 'model' designs

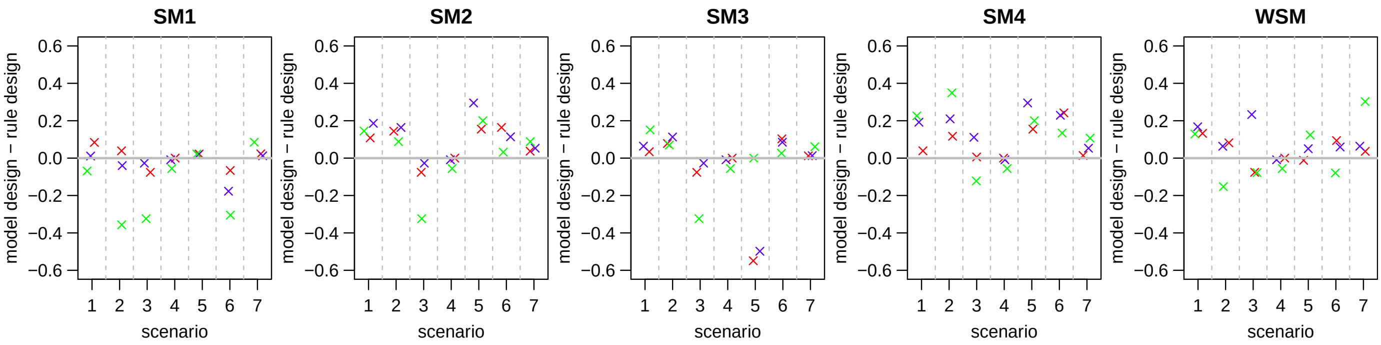

Positive values indicate that the 'model' design performed better than its 'rule' counterpart. 5pc, 10pc and 20pc designs are represented by red, blue and green symbols respectively.

Table S1: Operating characteristics under Scenario 2 (based on 1000 realisations)

	Dose									N ineffective‡	N below target§
	None	10mg	20mg	30mg	40mg	50mg	60mg	70mg	80mg		
True inefficacy		0.95	0.85	0.65	0.35	0.12	0.05	0.03	0.01		
5+5+5+5											
N per dose†		5.00	5.00		5.00				5.00	10.78	15.00
P(selection)⋆ - rule	0.05	0.00	0.00		0.12				0.83		
5%TIL											
N per dose†		2.00	2.00	0.06	3.10	2.51	2.75	0.08	5.51	5.17	9.67
P(selection)⋆ - rule	0.04	0.00	0.00	0.00	0.02	0.22	0.44	0.03	0.26		
P(selection)⋆ - model	0.03	0.00	0.00	0.00	0.01	0.19	0.52	0.09	0.16		
10%TIL											
N per dose†		2.00	2.00	0.29	3.78	3.93	2.55	0.33	3.12	5.75	12.00
P(selection)⋆ - rule	0.02	0.00	0.00	0.00	0.01	0.34	0.28	0.13	0.22		
P(selection)⋆ - model	0.00	0.00	0.00	0.00	0.02	0.39	0.39	0.18	0.02		
20%TIL											
N per dose†		2.00	2.03	0.77	5.91	3.80	0.99	0.06	2.45	6.72	14.51
P(selection)⋆ - rule	0.02	0.00	0.00	0.00	0.04	0.33	0.16	0.01	0.45		
P(selection)⋆ - model	0.00	0.00	0.00	0.00	0.15	0.59	0.23	0.03	0.00		

values for each trial design are means calculated over that design's set of realisations
⋆ the probability of selecting each dose (or no dose) to take forward to stage 2
† the number of subjects allocated to each dose
‡ the number of observed ineffective events
§ the number of subjects treated at doses below the target dose

Table S2: Operating characteristics under Scenario 3 (based on 1000 realisations)

	Dose									N ineffective‡	N below target§
	None	10mg	20mg	30mg	40mg	50mg	60mg	70mg	80mg		
True inefficacy		0.95	0.89	0.77	0.59	0.37	0.20	0.10	0.04		
5+5+5+5											
N per dose†		5.00	5.00		5.00				5.00	12.22	15.00
P(selection)⋆ - rule	0.19	0.00	0.00		0.01				0.80		
5%TIL											
N per dose†		2.00	2.00	0.01	2.13	0.57	1.78	0.18	9.33	5.98	8.67
P(selection)⋆ - rule	0.32	0.00	0.00	0.00	0.00	0.01	0.15	0.04	0.47		
P(selection)⋆ - model	0.32	0.00	0.00	0.00	0.00	0.01	0.15	0.13	0.39		
10%TIL											
N per dose†		2.00	2.00	0.09	2.38	1.36	2.67	1.10	6.40	6.52	11.60
P(selection)⋆ - rule	0.18	0.00	0.00	0.00	0.00	0.03	0.14	0.34	0.31		
P(selection)⋆ - model	0.05	0.00	0.00	0.00	0.00	0.03	0.16	0.48	0.28		
20%TIL											
N per dose†		2.00	2.00	0.15	2.97	2.82	3.08	0.67	4.29	7.52	13.71
P(selection)⋆ - rule	0.13	0.00	0.00	0.00	0.00	0.05	0.23	0.15	0.44		
P(selection)⋆ - model	0.00	0.00	0.00	0.00	0.00	0.11	0.41	0.35	0.12		

values for each trial design are means calculated over that design's set of realisations
⋆ the probability of selecting each dose (or no dose) to take forward to stage 2
† the number of subjects allocated to each dose
‡ the number of observed ineffective events
§ the number of subjects treated at doses below the target dose

Table S3: Operating characteristics under Scenario 4 (based on 1000 realisations)

	Dose									N ineffective‡	N below target§
	None	10mg	20mg	30mg	40mg	50mg	60mg	70mg	80mg		
True inefficacy		0.95	0.92	0.88	0.82	0.74	0.64	0.53	0.41		
5+5+5+5											
N per dose†		5.00	5.00		5.00				5.00	15.49	20.00
P(selection)⋆ - rule	0.93	0.00	0.00		0.00				0.07		
5%TIL											
N per dose†		2.00	2.00	0.00	2.01	0.03	0.07	0.00	11.89	10.33	18.00
P(selection)⋆ - rule	1.00	0.00	0.00	0.00	0.00	0.00	0.00	0.00	0.00		
P(selection)⋆ - model	1.00	0.00	0.00	0.00	0.00	0.00	0.00	0.00	0.00		
10%TIL											
N per dose†		2.00	2.00	0.00	2.03	0.07	0.26	0.04	11.60	10.40	18.00
P(selection)⋆ - rule	0.98	0.00	0.00	0.00	0.00	0.00	0.00	0.00	0.02		
P(selection)⋆ - model	0.98	0.00	0.00	0.00	0.00	0.00	0.00	0.00	0.02		
20%TIL											
N per dose†		2.00	2.00	0.00	2.06	0.23	0.30	0.16	11.24	10.63	18.00
P(selection)⋆ - rule	0.95	0.00	0.00	0.00	0.00	0.00	0.00	0.00	0.04		
P(selection)⋆ - model	0.89	0.00	0.00	0.00	0.00	0.00	0.00	0.02	0.09		

values for each trial design are means calculated over that design's set of realisations
⋆ the probability of selecting each dose (or no dose) to take forward to stage 2
† the number of subjects allocated to each dose
‡ the number of observed ineffective events
§ the number of subjects treated at doses below the target dose

Table S4: Operating characteristics under Scenario 5 (based on 1000 realisations)

	Dose									N ineffective‡	N below target§
	None	10mg	20mg	30mg	40mg	50mg	60mg	70mg	80mg		
True inefficacy		0.05	0.04	0.03	0.02	0.01	0.01	0.01	0.01		
5+5+5+5											
N per dose†		5.00	5.00		5.00				5.00	0.60	0.00
P(selection)⋆ - rule	0.04	0.56	0.15		0.16				0.09		
5%TIL											
N per dose†		2.00	10.08	1.28	2.37	0.16	0.06	0.00	2.05	0.59	0.00
P(selection)⋆ - rule	0.02	0.55	0.05	0.13	0.21	0.01	0.00	0.00	0.04		
P(selection)⋆ - model	0.00	0.00	0.75	0.16	0.05	0.02	0.00	0.00	0.00		
10%TIL											
N per dose†		2.00	10.97	0.75	2.13	0.13	0.00	0.00	2.01	0.65	0.00
P(selection)⋆ - rule	0.02	0.50	0.07	0.06	0.31	0.00	0.00	0.00	0.04		
P(selection)⋆ - model	0.00	0.00	0.86	0.10	0.01	0.02	0.00	0.00	0.00		
20%TIL											
N per dose†		5.87	7.43	0.48	2.19	0.03	0.00	0.00	2.00	0.65	0.00
P(selection)⋆ - rule	0.02	0.52	0.21	0.01	0.20	0.00	0.00	0.00	0.04		
P(selection)⋆ - model	0.00	0.52	0.41	0.05	0.02	0.00	0.00	0.00	0.00		

values for each trial design are means calculated over that design's set of realisations
⋆ the probability of selecting each dose (or no dose) to take forward to stage 2
† the number of subjects allocated to each dose
‡ the number of observed ineffective events
§ the number of subjects treated at doses below the target dose

Table S5: Operating characteristics under Scenario 6 (based on 1000 realisations)

	Dose									N ineffective‡	N below target§
	None	10mg	20mg	30mg	40mg	50mg	60mg	70mg	80mg		
True inefficacy		0.50	0.40	0.30	0.20	0.10	0.05	0.04	0.03		
5+5+5+5											
N per dose†		5.00	5.00		5.00				5.00	5.57	15.00
P(selection)⋆ - rule	0.14	0.00	0.02		0.29				0.56		
5%TIL											
N per dose†		2.00	2.48	1.91	4.71	2.67	1.28	0.05	2.89	3.94	13.78
P(selection)⋆ - rule	0.06	0.00	0.00	0.04	0.20	0.25	0.17	0.02	0.27		
P(selection)⋆ - model	0.02	0.00	0.00	0.06	0.21	0.33	0.27	0.08	0.04		
10%TIL											
N per dose†		2.00	2.86	2.71	5.05	2.17	0.71	0.07	2.42	4.35	14.79
P(selection)⋆ - rule	0.06	0.00	0.00	0.05	0.26	0.22	0.08	0.01	0.31		
P(selection)⋆ - model	0.00	0.00	0.01	0.13	0.30	0.34	0.16	0.04	0.02		
20%TIL											
N per dose†		2.01	4.08	3.78	4.86	0.91	0.18	0.03	2.15	4.88	15.64
P(selection)⋆ - rule	0.05	0.00	0.00	0.06	0.43	0.10	0.01	0.00	0.34		
P(selection)⋆ - model	0.00	0.00	0.04	0.32	0.38	0.20	0.04	0.01	0.00		

values for each trial design are means calculated over that design's set of realisations
⋆ the probability of selecting each dose (or no dose) to take forward to stage 2
† the number of subjects allocated to each dose
‡ the number of observed ineffective events
§ the number of subjects treated at doses below the target dose

Table S6: Operating characteristics under Scenario 7 (based on 1000 realisations)

	Dose									N ineffective‡	N below target§
	None	10mg	20mg	30mg	40mg	50mg	60mg	70mg	80mg		
True inefficacy		0.95	0.75	0.40	0.05	0.10	0.18	0.32	0.50		
5+5+5+5											
N per dose†		5.00	5.00		5.00				5.00	11.24	10.00
P(selection)⋆ - rule	0.97	0.00	0.00		0.02				0.00		
5%TIL											
N per dose†		2.00	2.00	0.20	3.17	0.70	0.26	0.04	9.63	8.56	4.20
P(selection)⋆ - rule	0.77	0.00	0.00	0.02	0.10	0.04	0.02	0.00	0.05		
P(selection)⋆ - model	0.77	0.00	0.00	0.00	0.11	0.06	0.03	0.01	0.01		
10%TIL											
N per dose†		2.00	2.01	0.45	3.71	0.34	0.14	0.03	9.33	8.48	4.46
P(selection)⋆ - rule	0.74	0.00	0.00	0.00	0.15	0.03	0.01	0.00	0.06		
P(selection)⋆ - model	0.73	0.00	0.00	0.00	0.17	0.07	0.01	0.01	0.00		
20%TIL											
N per dose†		2.00	2.08	1.09	3.31	0.51	0.26	0.07	8.69	8.56	5.16
P(selection)⋆ - rule	0.75	0.00	0.00	0.00	0.17	0.02	0.00	0.00	0.06		
P(selection)⋆ - model	0.64	0.00	0.00	0.02	0.23	0.04	0.03	0.01	0.02		

values for each trial design are means calculated over that design's set of realisations
⋆ the probability of selecting each dose (or no dose) to take forward to stage 2
† the number of subjects allocated to each dose
‡ the number of observed ineffective events
§ the number of subjects treated at doses below the target dose
